# Considerations of trial design and conduct in behavioral interventions for the management of chronic pain in adults

**DOI:** 10.1097/PR9.0000000000000655

**Published:** 2018-05-23

**Authors:** Sara N. Edmond, Dennis C. Turk, David A. Williams, Robert D. Kerns

**Affiliations:** a Pain Research, Informatics, Multimorbidities, and Education (PRIME) Center, VA Connecticut Healthcare System, West Haven, CT, USA; b Department of Psychiatry, Yale School of Medicine, New Haven, CT, USA; c Department of Anesthesiology and Pain Medicine, University of Washington, Seattle, WA, USA; Departments of d Anesthesiology; e Internal Medicine; f Psychiatry, and; g Psychology, University of Michigan, Ann Arbor, MI, USA; Departments of h Neurology and; i Psychology, Yale University, New Haven, CT, USA

**Keywords:** Chronic pain, Behavioral approaches, Clinical trials

## Abstract

**Introduction:**

A growing number and type of nonpharmacological approaches for the management of chronic pain have demonstrated at least modest evidence of efficacy, and for some, there is emerging evidence of their effectiveness in relatively large scale trials. Behavioral approaches are those that generally seek to promote adaptive behavioral change in the service of reducing pain and improving physical and emotional functioning and quality of life. Despite a substantial empirical literature supporting the clinical utility of these approaches, a large number of unanswered questions remain and clinical trials to answer some of these questions are needed. Although considerations for development and enactment of data-analytic plans are generally similar to those in pharmacological trials (eg, intent-to-treat, prespecifying outcomes and time points, and handling of missing data), there may be some important differences to consider when planning and conducting clinical trials examining these behavioral approaches.

**Objectives:**

The primary objective of this article is to describe some aspects of clinical trials for behavioral approaches for the management of chronic pain that requires special consideration.

**Methods:**

Important topics discussed include: (1) intervention development, (2) research design considerations (adequate and appropriate control and comparison conditions), (3) appropriate outcomes, (4) recruitment and sampling biases and blinding, (5) intervention fidelity and adherence, and (6) demographic and cultural considerations.

**Results and Conclusions:**

A number of methodological recommendations are made in the service of encouraging the conduct of high-quality research comparable with that performed for pharmacological and other medical interventions.

## 1. Introduction

In 2011, the Institute of Medicine (now the National Academy of Medicine) published its seminal report, *Relieving Pain in America*, calling for a cultural transformation in pain prevention, care, education, and research and recommending development of “a comprehensive population health-level strategy” to address these issues.^24^ In response to the report, the Department of Health and Human Services (HHS) created a National Pain Strategy that recommended expanded and sustained investment in research, informed by the biopsychosocial model and targeting development and implementation of evidence-based, integrated, multimodal, and interdisciplinary treatments for chronic pain.^45^

Although publications describing behavioral interventions to treat patients with chronic pain have a long history,^1,3,19,23,51,56^ pharmacological treatments continue to be the preferred treatment. This is despite the fact that the evidence supporting the efficacy of the treatments is modest at best for pharmacological as well as frequently provided invasive treatments (eg, nerve blocks, epidural steroids, and implantable devices).^52^ Moreover, these pharmacological and invasive treatments may have significant adverse effects. For example, over the past 2 decades, there has been a dramatic rise in the sale of prescription opioids in the United States and this has coincided with the rise of opioid-related adverse outcomes and overdose deaths.^13,14,33^ This has resulted in renewed interest in access to effective behavioral approaches designed to promote adaptive behavior change such as educational, psychosocial, exercise or movement, and some complementary approaches as alternatives and adjuncts to traditional medical and surgical interventions.^11^

Despite a substantial empirical literature supporting the clinical utility of behavioral approaches, a large number of unanswered questions remain. For example, few studies have been designed to directly compare these approaches with each other or in combinations with more traditional medical, surgical, or rehabilitation interventions. Some of these interventions are complex with multiple components (eg, cognitive-behavioral therapy [CBT]), delivered in different formats (eg, group, individual, and significant-other involvement), intensities, and duration (eg, number of sessions and variable time course); the mechanisms underlying positive outcomes, and the necessary and sufficient components are unknown. Although it is recognized that these approaches may be more effective for specific pain conditions, most clinical trials have limited their applications to conditions such as chronic low back pain, osteoarthritis, fibromyalgia, and headache, leaving it unclear whether the approach may be helpful for persons with other conditions (eg, neuropathic pain). Similarly, despite repeated calls from experts in the field, few studies have been designed with adequate power to examine important moderators of treatment effects such as age, sex and gender, race and ethnicity, or specific clinical characteristics.^16,53^ Of course, the same is largely true for virtually any clinical problem and intervention type. Moreover, as is true for pharmacological, medical, and surgical treatments, there are scant data as to the long-term benefits of these treatments.^52^

It is important to appreciate that although much of the guidance for clinical trials of pharmacological approaches described in other articles in this series and outlined in a number of U.S. Food and Drug Administration (FDA) and European Medicines Agency (EMA) guidance documents apply to the conduct of clinical trials for behavioral approaches (interested readers are encouraged to explore the FDA website and draft guidance on analgesic medication development),^54^ there are also a number of aspects of these latter trials that require particular attention.

The primary objective of this article is to describe special considerations for the design and execution of clinical trials for behavioral approaches for the management of chronic pain in the service of encouraging the conduct of high-quality research comparable with that performed for pharmacological and other medical interventions. The term “behavioral” is used throughout this article, as it is the commonly accepted term to characterize psychosocial interventions, modalities involving exercise and movement, and some complementary approaches. Although that is the focus of this article, much of what we discuss can be applied to other nondrug, nonsurgical, and other noninvasive interventions, such as public health, social work, occupational medicine, occupational therapy, physical therapy, and nerve stimulation and neuromodulatory, among others. Related, the conceptual, historical, and practical contexts for these types of interventions vary considerably. Having acknowledged these differences, we have focused our discussion on some of the specific considerations in research design and methodological issues that may be cross-cutting and generally applicable, and we have included examples from several of these domains. In the service of limiting our focus, the discussion focuses on efficacy trials, as opposed to effectiveness or implementation studies, although many of the concerns discussed are relevant to these later types of trials, as well. Important topics discussed include: (1) intervention development, (2) research design considerations (adequate and appropriate control or comparison conditions), (3) appropriate outcomes, (4) recruitment and sampling biases and blinding, (5) intervention fidelity and adherence, and (6) demographic and cultural considerations. Although these topics are important in all clinical trials, the challenges associated with addressing these domains in behavioral intervention trials may differ from approaches used in pharmacological and medical intervention trials.

Although considerations for development and enactment of data-analytic plans are generally similar to those in pharmacological trials (eg, intent-to-treat, prespecifying outcomes and time points, and handling of missing data), there may be some special analysis issues such as in the case of group randomized trials. Although adherence is a concern with all clinical trials, as noted, for treatments requiring patient acceptance such as nontraditional pharmacological and medical interventions, there are some particular challenges as the extent of participant demand may be higher for many behavioral approaches, particularly those that expect patients to engage actively in learning, practicing, and applying new skills and engaging in activities that have previously been avoided.

## 2. Intervention development

Standard pharmacotherapy trials seeking indications from the FDA and EMA include a set of 4 phases for determining formulation, dosing, safety, and efficacy. Phase 1 trials, usually the initial trials of a new compound in humans, focus on safety, pharmacokinetics, and maximum tolerated dosage. Phase 2 trials are commonly randomized clinical trials (RCTs) designed to compare the active compound with standard care or a placebo with the intent of establishing short-term risks, and preliminary estimates of the efficacy. Phase 3 trials are usually larger blinded RCTs with the intent of establishing the efficacy and monitoring for longer-term adverse events. Finally, phase 4 trials or postmarketing surveillance trials can involve thousands of individuals who have taken the drug with the intent of assessing longer-term safety and efficacy, as well as assessing real-world effectiveness and comparative effectiveness with other drugs in the market, cost-effectiveness, and long-term changes in health-related quality of life (HRQoL).

Common approaches for developing pharmaceutical interventions may not translate well when the intervention involves interpersonal and behavioral factors rather than a medication. As an alternative to the development standards of the FDA, the Stage Model of Behavioral Therapies was proposed in the 1990s to address the unique needs associated with developing behavioral interventions.^39^ This model proposes 3 stages. Stage I consists of 2 substages: (1a) therapy development and manual writing, and (1b) pilot and feasibility testing. Stage 1a is the most creative stage and begins with the identification of a theoretical foundation for the disorder, the postulated change process, and the targeted population for the intervention, specification of measures for evaluating the treatment, and clarification of how this treatment differs from other treatments already in existence.^7^ The product of stage 1a is a standardized therapist manual of treatment procedures, including initial ideas about appropriate dosing. The process by which the manual is developed at this stage relies heavily on the theoretical basis on which the treatment is based and clinical judgment (eg, content, format, and dose) rather than subjecting each decision to systematic empirical evaluation. Although some work at stage 1a can benefit by simply modifying preexisting established treatments, others may require assessment instrument development, needs assessments of the targeted population, or focus groups to identify relevant content. In addition, while developing treatment, it is important to consider any safety concerns related to appropriate dosing; for example, in exercise-based interventions, the key is often to find the line between too little and too much activity to improve functioning without leading to injuries or significant exacerbation of pain. It may be worth noting that a growing number of funding agencies and organizations (eg, National Institutes of Health [NIH]) now expect to fund “planning grants” that generally support stages 1a and 1b, for either pharmacological or nonpharmacological intervention research.

Stage 1b involves conducting an initial pilot trial of the new intervention or application. The intent of this stage is to provide initial estimates of efficacy, patient acceptance, feasibility of delivery, and optimal context, to develop procedures for identifying, training, and supervising therapists, and to identify relevant outcomes and relevant comparators.^39^ The goal for this stage is to demonstrate that the treatment can be delivered and has merit when delivered under “optimal” conditions—this helps to justify larger studies of the treatment.

Stage 2 focuses on conducting RCTs of the intervention based on a detailed manual and provider training in a standardized and structured environment as a means of supporting the treatment's efficacy. Stage 2 studies can also be used to determine mechanisms of action.

Stage 3 moves beyond the efficacy to evaluate “effectiveness” in real-world settings in contrast to “efficacy” in a controlled environment. For example, effectiveness trials may be designed to assess the impact of different types of practitioners on the therapy, evaluate whether the intervention retains its benefit for less well-screened patients, and examine benefit in the setting of clinical practice. Cost-effectiveness issues can also be addressed in stage 3.^39^ Once the efficacy is established, subsequent stage 3 studies can also be used to dismantle the multiple components of interventions (interventionist attention, patient expectations, theoretical content, duration, and patient adherence) in search of the “active” elements (ie, mechanisms) as well as mediators and moderators of change. In contrast to drug trials, in which dose is often explored earlier, the concept of optimal dosing of behavioral interventions is most often explored once the efficacy is established. Given that toxicity is not typically an issue for psychological and behavioral interventions (although symptom exacerbation and precipitation of emotional distress and associated problems might be raised with some interventions), dosing is not often a critical safety issue. However, intensity is still important to explore, as it may be critical for efficacy and treatment engagement. In summary, the stage model of intervention development is similar to the drug development model in that it applies rigorous methodological standards and approaches at each stage but modifies the intent of each stage to be better matched to the characteristics of behavioral interventions, including a more explicit focus on the theoretical basis for the intervention (Table 1).

**Table 1:**
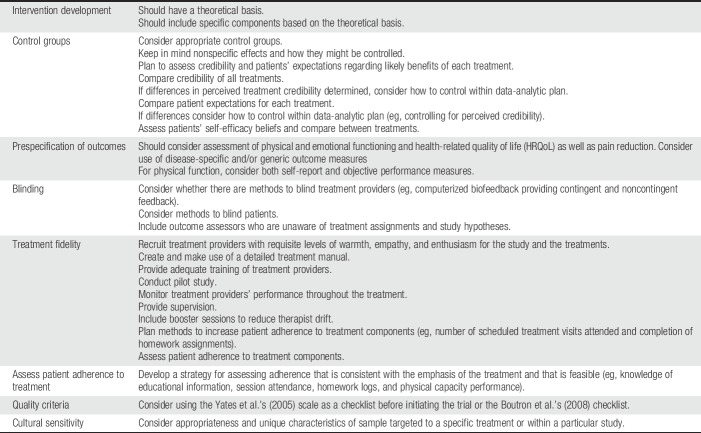
Key consideration in behavioral clinical trials for patients with chronic pain.

## 3. Research design considerations

### 3.1. Control and comparator groups and the placebo effects

In all RCTs, choosing a comparator group is an important aspect of trial design. There are several things to consider when choosing comparator groups for clinical trials, including scientific, clinical, practical, budgetary, and ethical considerations; this section will focus primarily on the scientific considerations related to choosing a comparator group.

In pharmacological trials, the gold-standard trial design is a double-blind placebo-controlled RCT. In double-blind placebo-controlled designs, pharmacologically inert agents are often used to control for the effects of being given a medication. In some designs, placebo agents that may mimic some side effects of the active drugs are used (ie, active comparators), which helps to mask the fact that the alternative being given is a placebo; however, ethical concerns have been raised about the use of such active comparators (see Rowbotham's article in this series). Furthermore, in this trial design, because of the double-blinded nature, clinical attention can be equivalent for both groups, allowing researchers to isolate the effect of the biological agent, controlling for both the placebo effect (ingesting medicine and beliefs about what that means) and clinical attention (eg, from study staff). Similarly, in comparative trials of 2 or more pharmacological agents, medications with different biological agents can be compared without much risk of compromising the double-blind nature of the study, and the placebo effect of being enrolled in a study and receiving clinical attention can be equivalent across treatment conditions. Cross-over trials that consist of 2 phases where patients receive each of the alternative treatments with a “wash out” period in between to eliminate the drug effects have some particular advantages, as patients serve as their own controls and this may reduce the sample size required. For behavioral treatments, cross-over trials are more problematic or impossible because for many educational, psychological, and exercise-based treatments, it is impossible to eliminate (“wash out”) the continuing effects of the treatment between phases.

In behavioral intervention research, the gold-standard trial design is less clear, particularly about the ideal comparator or control group.^20,31^ For that reason, behavioral trials have used a wide array of comparator groups in attempt to mimic placebos and the amount of attention included in pharmacological trials. To understand how to best choose a control or comparison group for a behavioral RCT, it is important to consider the purpose of the trial, trial design, and comparator, as well as the advantages and disadvantages of various alternative comparator conditions.

When the primary purpose of a behavioral trial is to determine whether an active treatment is more efficacious than no treatment (or treatment as usual [TAU]), a randomized design and a control group are needed to control for potentially confounding variables, including classic threats to internal validity (eg, selection bias or regression toward the mean) or other rival explanations of effects (eg, the placebo effect or attention). Identifying an ideal control group for this situation is challenging, and depends on what rival explanation investigators are concerned about and why ruling out that rival explanation is important.

Although pharmacological trials typically use comparator or control groups that allow for a double-blind design, this is more challenging with behavioral interventions. Using a design that assigns participants to no treatment, a wait list, or TAU will eliminate the possibility that participants and treatment providers are blind to the intervention. In addition, these types of comparator groups often involve a different amount of participant contact with treatment providers across arms, raising the question of whether differences in clinical attention account for group differences. For this reason, attention control conditions are common in behavioral intervention research.

Unfortunately, attention control conditions also have a number of limitations, and there are no gold standards for developing these types of conditions. First, although clinical attention may be a meaningful and active ingredient for most interventions, which may influence one's power to detect effects or the types of conclusions one can draw about the efficacy of the intervention, attention can be particularly potent in many behavioral treatments that are dependent or predicated on significant involvement of the therapy provider. Second, as researchers attempt to design attention controls that seem credible to participants, these control groups may contain unintentionally “active” ingredients; this is particularly true when researchers may not clearly know all the possible mechanisms of action in their active conditions. For this reason, it is important to consider carefully the scientific question one wishes to answer and use that as a guide in choosing comparator groups. Despite these challenges, some researchers have shown differences in outcomes with the use of attention control conditions. For example, 1 trial comparing CBT with supportive psychotherapy for vulvodynia found significantly greater improvements in some measures of physician-reported pain and sexual functioning for the CBT condition as compared to the supportive psychotherapy condition,^29^ although both groups improved on measures of self-reported pain and emotional functioning. When examining the efficacy of acupuncture, sham acupuncture with superficial entry or sham needle placements has been used as comparator group, with some randomized trials finding benefit for acupuncture as compared to sham acupuncture on measures of pain-related disability and patient functioning;^12^ however, some studies have also reported that participants are able to identify sham acupuncture with up to 83% accuracy.^57^ Similarly, there have been trials of biofeedback comparing respiratory feedback with noncontingent feedback^26^ and trials of physical therapy comparing protocols including patellar taping with protocols including placebo taping.^15^

Several types of control conditions have been described in the literature that may be useful in behavioral trials. Usual care or TAU conditions offer the opportunity to answer the question “would adding my intervention to existing usual care significantly improve patient outcomes?” One drawback of usual care or TAU control conditions is the lack of oversight of what happens in usual care settings. Thus, if providing increased oversight of standardization of TAU control conditions is possible, it may be beneficial. For some research questions, an enhanced or minimally enhanced TAU condition (eg, providing written education to patients or providers) may prove useful; in some situations, constrained TAU (eg, prohibiting the use of a certain type of medication during study participation) may be reasonable. Furthermore, rather than allowing TAU to continue somewhat outside the control of the investigator, if standardized treatment regimens or standards of care exist, offering these interventions to control condition patients may provide a useful comparator group; however, these types of control conditions may be likely to improve outcomes more than traditional usual care, leading to reduced effect sizes and the need for a larger sample sizes. One final consideration is the issue of treatment credibility. Optimally, control and comparison conditions will not differ significantly in terms of participant perceptions of credibility relative to the experimental condition.^17^ Measuring participants' expectations before treatment and perceived treatment credibility after treatment is one way to address this concern.

When choosing a comparator group, considering the stage of trial design along with the scientific question is useful. For example, stage 2 trials focused on demonstrating efficacy may want to consider usual care as routine or minimally enhanced usual care conditions to answer the question “is my treatment better than no treatment, or the treatment typically encountered in routine care?” However, if differential amounts of attention are a major concern for researchers, an attention control condition may be warranted, although it is important to avoid accidentally including active ingredients in these control conditions included in the trial. In stage 3 trials that move beyond efficacy and focus more on effectiveness, usual care or minimally enhanced usual care may be ideal, given that they mimic more real-world settings. These trials may also begin to consider questions related to comparative effectiveness, in which 2 active treatments are compared. In summary, when conducting an RCT, whether pharmacological, behavioral, or otherwise, no control condition is perfect. Investigators must thoughtfully consider a range of factors when choosing comparator groups, such as the purpose of the comparator, the purpose of the trial, nonspecific effects, treatment credibility, and patients' expectations of treatment.

## 4. Outcomes in clinical trials

Traditionally, the primary and often sole outcome in chronic pain treatment studies has been the alleviation of pain. However, there has been growing recognition that functional outcomes (ie, improvement in physical and emotional function) and HRQoL may be at least as important.^43,48^ This is especially important when there is recognition that in the vast majority of cases, currently available treatments, even when they may reduce pain intensity significantly, rarely are capable of completely eliminating pain. This reality can be readily observed in evaluating published reports of trials testing the efficacy of medications with putative analgesic properties (eg, opioids, nonsteroidal anti-inflammatory drugs, anticonvulsants, and antidepressants).^52^ Typically, in these studies, an entry criterion for enrollment is a self-report rating of pain ≥4 on a 0 to 10 numerical rating scale, with 4 connoting moderate pain. Examination of the outcomes of this large body of research reveals that even when treatment effects on pain reduction are statistically significant, for the vast majority of the sample, on average pain remains ≥4. Thus, even “successfully” treated patients, at the end of the trial, would continue to report pain to be sufficiently high so as to make them eligible to enroll in another clinical trial!

Investigators, clinicians, and patients may believe that if pain is reduced, even when not eliminated, there will be a significant accompanying improvement in physical and emotional functioning and HRQoL. However, there is a large literature demonstrating that there is not a high association between pain, functioning, and HRQoL. That is, reducing pain does not seem sufficient to improve these other important aspects of patients' lives. In contrast to conventional pharmacological treatments, for some behavioral treatments, pain is not considered the primary outcome. Rather, the emphasis of some studies has been directed toward improvement in functioning and HRQoL. This emphasis has resulted in recommendations that improvement in functioning in various domains of life should be considered as an important outcome as pain reduction. For example, the Initiative on Methods, Measures, and Pain Assessment in Clinical Trials (IMMPACT) has recommended assessing outcomes representing 6 core domains: (1) pain, (2) physical functioning, (3) emotional functioning, (4) participant ratings of improvement and satisfaction with treatment, (5) symptoms and adverse events, and (6) participant disposition.^48^

Investigations of patient preferences for outcomes confirm that although patients view pain reduction as an important outcome for any treatment, they also consider other important outcomes, especially those related to improvement in physical functioning. One large patient survey specifically identified emotional well-being, fatigue, weakness, sleep-related problems, and enjoyment of life as important outcomes from patients' perspectives.^49^

A substantial literature exists related to the methods and measures that should be considered as outcomes in all clinical trials evaluating the efficacy and effectiveness of pharmacological as well as behavioral studies.^48^ There are numerous published reviews and critiques of measures to assess physical and emotional functioning and HRQoL as well as pain.^43,50^ Important considerations raised are whether to use generic measures or measures that are specific to the diagnoses of the participants in the clinical trials. For example, IMMPACT recommendations were that for primary outcomes, when available, measures that are disease specific should be considered, whereas generic measures should be considered, at least as secondary outcomes, when there is an interest in comparing effects of treatments across multiple disorders.^47^ IMMPACT suggested several generic measures that might be considered.^18^ Taylor et al.^43^ provided a comprehensive review of a broad array of disease-specific measures that might be considered as appropriate outcomes when assessing physical and emotional functioning along with more general measures of emotional, work-related, and social functioning. These authors also raised the importance of acknowledging that physical function or activity can be assessed using self-report measures and other objective measures (eg, actigraphy and performance). They noted that although self-report measures of activity and objective measures of physical functioning are both important, they are not equivalent and should be considered as complementary as they are providing different information about patient functioning.

Regardless of what constructs are selected as the primary outcome(s), it is essential that investigators prespecify primary and secondary outcomes, providing the rationale for why these were selected, as a means to reduce switching of outcome, or reporting additional outcomes on a post hoc basis. This, of course, is not unique to clinical trials of behavioral interventions, although it may be of particular importance given that behavioral interventions may be interested in a range of outcomes beyond pain intensity including pain-related physical and emotional functioning and quality of life. Furthermore, researchers should be mindful of the tension between reporting an extensive number of recommended measures and the importance of not overreporting outcomes that were not prespecified.

## 5. Recruitment, sampling, and blinding

The purpose of an RCT is to evaluate the “true” effect of an experimental intervention. To properly interpret such an effect, one must minimize potential biases that can be introduced by recruitment methodology (ie, minimizing threats to external validity) and the trial methodology itself (ie, minimizing threats to internal validity). There are a number of issues that are important to consider.

For example, the issue of potential recruitment and sampling biases and strategies to overcome them is an important consideration. Although concerns about recruitment bias are appropriate for all interventions described in this series and clinical trials in general, it is reasonable to anticipate that sampling bias is a particular problem for behavioral treatments because persons participating in these trials must be interested and willing to receive what they may perceive as nontraditional approaches, especially ones that may require greater participant burden and responsibility for actively engaging in the intervention. Referral biases for psychological treatments may be commonplace as well because providers may have a tendency to refer patients for these trials who are highly distressed or those with substance abuse problems, whereas others who might benefit are less likely to be referred. Given these possibilities, concerns about generalizability (external validity but not internal validity) need to be addressed (eg, through enhancing advertising strategies, recruitment, and referral methods to reach typically hard-to-enroll patients; by minimizing inclusion and exclusion criteria, or by reducing participant burden to encourage participation by those who typically fail to volunteer to participate).^27^

In addition to referral bias, additional potential biases in clinical trials arise from 4 primary sources: selection bias (eg, biased allocation to the treatment or comparator groups), performance bias (eg, clinicians favoring or providing unequal care to 1 of the treatment arms), detection bias (eg, unequal assessment of outcomes), and attribution bias (eg, unequal handling of protocol deviations and loss to follow-up).^25^ One method of addressing 2 of these sources of bias (ie, performance and detection bias) is the use of “blinding.”^35^ Blinding is essentially concealing knowledge of what treatment is being given to whom. Blinded individuals can be study participants, clinicians, assessors of outcomes, or all these groups. Each of these groups of individuals is capable of biasing the estimate of the “true effect” of the treatment. For example, if participants know what intervention they are receiving, their physical, psychological (eg, expectations), or behavioral responses (eg, adherence and retention) could be influenced either in favor of or against the experimental or comparator intervention. When treatment providers are unblinded, there is the potential of their favoring one treatment over another, inadvertently communicating the identity of the treatment to blinded study participants, unequally adding cointerventions or adjusting dosages, and unequally withdrawing participants or encouraging retention in the trial. Unblinded assessors can similarly influence outcomes when they know treatment assignment by unwittingly or intentionally favoring or negatively influencing outcome ratings. Although hard outcomes such as death are relatively immune to this source of bias, more subjective outcomes (eg, quality of life, pain, functional status, and pain and disability severity grading) can be particularly vulnerable to this effect.^35^

Currently, the industry standard for an RCT is the use of double-blind methodology.^30^ Although this methodology is easily applicable to pharmaceutical trials (eg, disguising an inert substance), it is more difficult to blind behavioral interventions in this way (eg, therapists and participants must know what type of treatment is being delivered to engage in that treatment). Although it is more difficult to double-blind behavioral treatments, it is possible, at least in some instances. For example, biofeedback can be computerized so as to provide contingent and noncontingent feedback. When not possible to incorporate a double-blind methodology, rather than assuming that all behavioral studies are of lower quality because of a limited ability to blind, it is preferable to blind when appropriate and then evaluate the study's internal validity based on the overall merits of the study. It is important to resist the temptation to uncritically extrapolate from pharmacological trials and insist on a specific checklist of methodologies for evaluating the merits of all clinical trials. For example, rigorous allocation concealment (ie, the prevention of selection bias by keeping the treatment assignment sequence private before assignment) is also an important part of reducing bias and can be easily applied to most behavioral clinical trials.^41^ Thus, when evaluating a study, a thoughtful evaluation of the study in its entirety is needed along with better descriptions of what actually occurred and how safeguards to reduce bias were actually implemented.^31^ Some suggestions have been proposed as ways to evaluate the quality of methodologies incorporated within a clinical trial for behavioral treatments, such as precise reporting of both experimental and comparator condition methodology, details about treatment standardization, treatment provider training, characteristics, and adherence, and if and how any blinding was incorporated in the study design.^10,58^

In cases where blinding of a behavioral study can be accommodated, it is most likely to involve blinding of the outcomes assessor.^41^ This usually takes the form of using a centralized assessment of the primary outcome.^9^ Examples include using blinded research staff to administer and score self-report questionnaires, videotaping behaviors such as walking or spouse interactions to be later scored by trained raters, or using photographs of physical outcomes such as wound healing to be later scored by independent raters. For some studies, participants and providers can be blinded by being blind to the study hypothesis.^9^ For example, the intent of the trial may be to study adherence or self-efficacy rather than the assumed target of the intervention (eg, symptom reduction). In such cases, knowledge of what intervention was being offered would not reveal the intent of the study (eg, adherence) and therefore would be less likely to threaten the internal validity of the study.

An important caveat is that systematic reviews and meta-analyses often express their conclusions in very guarded terms, referring in particular to methodological shortcomings with the potential to bias results, and to the quality of trials. However, most of the commonly used quality scales were devised for drug trials and not behavioral treatments, with obvious differences, such as the impossibility of blinding the treatment provider delivering a behavioral intervention, or of providing a “placebo” control condition that is indistinguishable from the active intervention (see Yates et al.^58^ for a discussion of these issues and a scale for assessing psychological trials and Boutron et al.^10^ for nonpharmacological treatments more generally).

In behavioral treatment outcome studies, traditional blinding of providers and patients poses significant problems as does establishing appropriate control groups.^42^ Behavioral trials may be penalized in meta-analyses, systematic reviews, and treatment guidelines because they are unable to include double blinding.^46^ Although these biases could lead to larger effect sizes and erroneously make these treatments look more effective, it is important not to discard findings solely because they are not double blinded.

## 6. Intervention fidelity

Knowledge of the quality of intervention delivery—intervention fidelity—is critical for interpreting the results of a clinical trial. Two broad types of fidelity are relevant: treatment integrity (ie, was the treatment implemented as intended) and treatment differentiation (ie, do comparators differ from the experimental treatment along with critical theoretical dimensions).^7^ These types of fidelity aid study interpretation in cases where an intervention is found to be beneficial. For example, when found to be beneficial, it must be known whether the intervention was actually delivered according to protocol or whether extraneous unstandardized factors may have accounted for the benefit (eg, addition of unintended external content/procedures or omission of some protocol elements). Conversely, if a new treatment is not found to be beneficial, evidence related to fidelity can aid in determining whether the intervention itself was ineffective or whether the internal validity of the study was compromised.

Five components of treatment fidelity were identified by the 2004 NIH Behavior Change Consortium as being relevant to psychosocial interventions: design, therapist training, treatment delivery, participant receipt of the intervention, and treatment enactment.^4^ To better understand fidelity, each component will be briefly described.

### 6.1. Design

Some procedures to enhance fidelity can be implemented before initiating the study, while the study is being designed. “Fidelity in design” refers to ensuring that the experimental treatment under study adequately tests the underlying theory or related clinical processes. As mentioned above in stage 1 of “intervention development,” each intervention should have a theoretical grounding with a purported “active ingredient” accounting for the particular intervention's benefit. Study planners can boost fidelity by insuring that extraneous confounding or contaminating factors are minimized when considering the treatment, comparator treatments, and control groups within the design. For example, if cognitive restructuring is a purported active ingredient of an experimental treatment, comparators should be devoid of this ingredient.

Other design-related fidelity considerations include standardizing the dose of treatment within each condition (eg, each participant receives the same number of treatments, frequency of treatments, and length of contact within a treatment arm), and that dosing approximates equivalence across conditions. Study planners should also anticipate loss or turnover of trained providers. If a well-trained therapist leaves the study, the integrity of the treatment could be compromised by suboptimally trained replacements. Training back-up pools of providers can help with this issue.^4^

### 6.2. Training

For a treatment to be delivered as intended, at a minimum, providers need to have received the same training and access to the same set of therapeutic skills. This training helps to minimize differential outcomes based on the provider rather than the treatment itself. Provider training should be standardized and often involves the use of provider manuals, training in groups, and role-playing with standardized mock patients. Before initiating the study, providers should meet well-defined performance criteria that can be assessed with written knowledge-based examinations, observation in role-play, or both. Given the tendency for provider skills to drift over time, it is recommended that regular supervision of therapists occurs and that booster sessions be used to recalibrate providers to the original standardized protocol.^4^ Once the study begins, supervised sessions can be helpful in addressing real-world issues that arise and can insure that proposed solutions are consistent with the protocol. Clear definitions of provider education and qualifications can also help to insure fidelity in training.^38^ Finally, the relative pros and cons of using separate providers for each condition vs having all providers deliver all conditions should be considered. Although cross-nesting interventionists may increase concerns about strict adherence to conditions (eg, concerns about cross-contamination of conditions), this can be managed by continuing fidelity monitoring throughout the trial, while using separate interventionists for each condition introduces the problem of interventionist characteristics influencing treatment outcomes.

### 6.3. Delivery

A well-trained provider may or may not deliver the intervention in accord with the protocol. Time constraints may lead to omissions of content and previous therapeutic experiences may lead a therapist to insert extraneous content and deviations into the protocol. Interviewing potential applicants before initiating the study can help insure the presence of desired therapist characteristics (eg, empathy, warmth, and enthusiasm) so that these characteristics are evenly distributed across conditions.^38^ Audio or videotaping sessions can also help monitor provider delivery fidelity through the use of content checklists as well as the use of supervision forums where sessions can be reviewed.^4^

### 6.4. Receipt of treatment

A well-trained prescriber can select, instruct, and administer an efficacious medication, but if the patient never takes it, it will not be helpful. Similarly, in behavioral interventions, fidelity checks need to be made both on the provider side as well as on the side of the patient recipient. That is, it is important to know whether participants actually comprehend what the provider is providing and what the patient's responsibilities are when required (eg, home practice). If participants lack comprehension, an otherwise beneficial intervention will fail to show any effect. Not only must participants understand the content, they must have the ability and willingness to use the skills being discussed when required. For example, in an aquatic exercise intervention, participants may comprehend the importance and potential aerobic benefits of such exercise but may not have access to a pool. Pretest and posttest knowledge questionnaires, review of homework, and self-monitoring logs can assist with this form of fidelity check. Typically, receipt of treatment fidelity monitors for both comprehension and initial use of the strategies during treatment delivery (eg, narrative notes on participation, comprehension, and effort on homework).^38^

### 6.5. Enactment

Enactment refers to whether participants use the active ingredient of the treatment, as prescribed, at the appropriate time in real-life situations. Use of the skill in real-life situations would assume that (1) the skill had been appropriately taught, (2) comprehended, and (3) learned sufficiently to be acted on when appropriate. These aspects of enactment are often evaluated through the knowledge-based assessments, use of self-report logs, staff observation, and interviews. Enactment fidelity is typically assessed posttreatment or during a follow-up period. In 2011, Borelli published a 30-item checklist for implementing and reporting on all 5 components of fidelity in clinical trials.^7,8,37^

## 7. Intervention adherence

The term “adherence” (compliance) again comes from pharmaceutical clinical trial terminology referring to the extent to which a patient follows medical instructions (eg, pill taking).^40^ In contrast to the conventional expectation of passive patient acquiescence to providers' instructions, adherence implies a more active, voluntary collaborative involvement of a patient in a mutually acceptable course of behavior to produce a desired preventive or therapeutic result. The concept of adherence can be and has been expanded to include aspects of behavioral treatments, but this latter application significantly complicates the original meaning.^40^

Adherence can refer to the investigator and therapist adhering to the treatment protocol and to the patient adhering to the treatment recommendations (eg, attending scheduled appointments and completing assigned tasks). In addition, the therapist might include audiotaping of the treatment session or direct observation by a third party to determine whether the protocol and content were followed as specified in a treatment manual. There are a number of different ways to assess patient adherence, which may include measures of treatment attendance, actual pill consumption (date and time), urine and blood assays, and measures of actual participation in water aerobics (date and time).

A patient may believe that the intervention likely has some benefit, but they may lack the belief that they are competent to make use of the content, that is, they may have low self-efficacy^2^ or high levels of fear of performance of some physical exercises because they believe that they will injury themselves or performance will exacerbate their pain.^55^ In studies where both enactment and adherence are poor, it becomes difficult to discern whether the enactment was poor (ie, poor internal reliability) or whether the overall treatment was simply nonefficacious.^4^

In addition to methods to assess adherence described in the previous section of this article, homework logs and attendance at therapy sessions can also be used to assess patient adherence. For exercise-based and activity-based treatments, assessment of changes in physical capacity or functioning can be used as a surrogate for adherence.

## 8. Demographic and cultural considerations

Demographic and cultural considerations, while important in all research, play an important role in the design of clinical trials involving behavioral approaches to the management of chronic pain. In addition to the need to consider demographically and culturally sensitive recruitment strategies, behavioral approaches may also consider adapting intervention content to be more relevant to special populations.

Drawing on the psychotherapy literature, it has been hypothesized that evidence-based psychological treatments may be more effective when they are compatible with patients' cultural patterns and world views.^6,44^ This may also be the case for behavioral approaches to the management of chronic pain. Cultural adaptation may improve treatment engagement, retention, relevance, and outcomes.^21,32^ Relatively few studies to date have explicitly examined the efficacy of culturally adapted interventions to manage chronic pain.

Tailoring behavioral interventions to be relevant to the patient population can take a number of forms. For example, the language of the intervention is an important consideration. If interventions are not available in a language that can be understood by a patient, it may be difficult or impossible to use. Culturally appropriate translations are essential to ensuring that interventions can be used with non-English–speaking populations. Furthermore, it may be important to consider adapting languages within English-speaking populations to reflect dialects and cultural norms of the population being treated. A handful of research studies have reported on adaptations to CBT for chronic pain materials for specific patient populations. For example, Reid et al.^36^ developed and piloted a tailored protocol for older adults with chronic low back pain. Tailoring methods included the use of larger font on handouts and the use of age-appropriate examples. Thorn et al.^47^ tailored a CBT for chronic pain protocol for a rural audience who was majority African American, low socioeconomic status, and low literacy. Adaptations to materials included revising content to be written in active voice with simple sentence structures at a fifth-grade reading level, increasing the use of white space, and using culturally appropriate graphics. This research group interviewed key informants and conducted focus groups with their target population to modify materials before conducting a randomized trial.^28^

Cultural knowledge and metaphors, including symbols, idioms, and concepts, should also be considered in intervention development when possible. For example, in CBT for chronic pain, a popular metaphor to describe the difference between acute and chronic pain is the “check engine light” metaphor, in which the check engine light of a car is used as a metaphor for the experience of pain. For populations who have little experience driving or do not own a car, more suitable metaphors and analogies may be needed.

Treatment methods may also need to be modified based on demographic and cultural factors. For example, although many behavioral treatments are designed to be delivered to patients individually, some cultures value collectivism and family involvement. Considerations of involving family members in treatment (eg, allowing them to attend sessions) may improve treatment engagement and the efficacy in these cases. Similarly, considerations of the timing or format of delivery (eg, weekly vs every other week; in person vs using technology-assisted delivery) may enhance treatment engagement for populations who may otherwise find it challenging to engage in behavioral interventions (eg, because of work or childcare obligations). For example, there is a growing body of work examining ways to use technology to adapt CBT-CP, such as the use of interactive voice response technology^22^ or web-based platforms.^5^

## 9. Conclusion

Interest in development and evaluation of novel nonopioid pharmacological and particularly behavioral approaches to pain management is growing. Conducting trials for behavioral approaches for pain management engenders a number of issues that diverge from traditional analgesic studies. In Table 1, we have outlined several key issues that should be considered in designing a clinical trial of such approaches. This article highlights these and other issues and offers a discussion of possible options for addressing and resolving them. In particular, discussed are issues related to stages of intervention development, research design, adequate and appropriate control and comparison conditions, outcomes, including the importance of a priori specification of primary outcomes, recruitment and sampling biases and blinding, treatment specificity and fidelity, patient acceptance of treatment and adherence, and cultural sensitivity. Investigators contemplating a clinical trial of a novel pain management approach are encouraged to consider these issues and decisions that may be relatively specific to the conduct of trials of behavioral interventions as well as the additional guidance for designing and conducting any clinical trial offered in this series.

## Disclosures

D.A. Williams is a consultant for Community Health Focus, Inc., D.C. Turk reports grants from ACTTION public-private partnership, which has received research contracts, grants, or other revenue from the U.S. Food and Drug Administration, multiple pharmaceutical and device companies, philanthropy, and other sources (list of current sponsors can be found at www.acttion.org/partners), during the conduct of the study; personal fees from Flexion, personal fees from Pfizer, personal fees from St. Jude, outside the submitted work; and Editor-in-Chief, Clinical Journal of Pain. R.D. Kerns is Senior Editor, Pain Medicine, and receives an honorarium for this service. S.N. Edmond has no conflicts of interest to declare.

This work was supported by the VA Health Services Research and Development Service Center of Innovation (CIN 13-407).

The views expressed in this article are those of the authors and no official endorsement by the FDA or the pharmaceutical and device companies that provided unrestricted grants to support the activities of the ACTTION public-private partnership should be inferred. Financial support for this project was provided by the ACTTION public-private partnership, which has received research contracts, grants, or other revenue from the FDA, multiple pharmaceutical and device companies, philanthropy, and other sources.

The views expressed in this article are those of the authors and do not necessarily represent the position or policy of the Department of Veterans Affairs or the U.S. government.

## References

[1] Andersson SA Holmgren E On acupuncture analgesia and the mechanism of pain. Am J Chin Med (Gard City N Y) 1975;3:311–34. 173171 10.1142/s0192415x75000396

[2] Bandura A Self-efficacy: the exercise of control. New York: Freeman, 1997.

[3] Bates JA Nathan PW Transcutaneous electrical nerve stimulation for chronic pain. Anaesthesia 1980;35:817–22. 6969555 10.1111/j.1365-2044.1980.tb03926.x

[4] Bellg AJ Borrelli B Resnick B Hecht J Minicucci DS Ory M Ogedegbe G Orwig D Ernst D Czajkowski S Enhancing treatment fidelity in health behavior change studies: best practices and recommendations from the NIH Behavior Change Consortium. Health Psychol 2004;23:443–51. 15367063 10.1037/0278-6133.23.5.443

[5] Bennell KL Nelligan R Dobson F Rini C Keefe F Kasza J French S Bryant C Dalwood A Abbott JH Hinman RS Effectiveness of an internet-delivered exercise and pain-coping skills training intervention for persons with chronic knee pain: a randomized trial. Ann Intern Med 2017;166:453–62. 28241215 10.7326/M16-1714

[6] Bernal G Jimenez-Chafey MI Domench Rodriquez MM Cultural adaptation of treatments: a resource for considering culture in evidenced-based practice. Prof Psychol Res Pract 2009;40:361–68.

[7] Borrelli B The assessment, monitoring, and enhancement of treatment fidelity in public health clinical trials. J Public Health Dent 2011;71(suppl 1):S52–S63. 10.1111/j.1752-7325.2011.00233.x PMC3074245 21499543

[8] Borrelli B Sepinwall D Ernst D Bellg AJ Czajkowski S Breger R DeFrancesco C Levesque C Resnick B Orwig D A new tool to assess treatment fidelity and evaluation of treatment fidelity across 10 years of health behavior research. J Consult Clin Psychol 2005;73:852–60. 16287385 10.1037/0022-006X.73.5.852

[9] Boutron I Guittet L Estellat C Moher D Hróbjartsson A Ravaud P Reporting methods of blinding in randomized trials assessing nonpharmacological treatments. PLoS Med 2007;4:e61. 17311468 10.1371/journal.pmed.0040061 PMC1800311

[10] Boutron I Moher D Altman DG Schulz KF Ravaud P; CONSORT Group. Methods and processes of the CONSORT Group: example of an extension for trials assessing nonpharmacological treatments. Ann Intern Med 2008;148:295–309. 18283201 10.7326/0003-4819-148-4-200802190-00008-w1

[11] Chang KL Fillingim R Hurley RW Schmidt S Chronic pain management: nonpharmacological therapies for chronic pain. FP Essent 2015;432:21–6. 25970869

[12] Cho YJ Song YK Cha YY Shin BC Shin IH Park HJ Lee HS Kim KW Cho JH Chung WS Lee JH Song MY Acupuncture for chronic low back pain: a multicenter, randomized, patient-assessor blind, sham-controlled clinical trial. Spine (Phila Pa 1976) 2013;38:549–57. 23026870 10.1097/BRS.0b013e318275e601

[13] Compton WM Boyle M Wargo E Prescription opioid abuse: problems and responses. Prev Med 2015;80:5–9. 25871819 10.1016/j.ypmed.2015.04.003

[14] Compton WM Volkow ND Major increases in opioid analgesic abuse in the United States: concerns and strategies. Drug Alcohol Depend 2006;81:103–7. 16023304 10.1016/j.drugalcdep.2005.05.009

[15] Crossley K Bennell K Green S Cowan S McConnell J Physical therapy for patellofemoral pain: a randomized, double-blinded, placebo-controlled trial. Am J Sports Med 2002;30:857–65. 12435653 10.1177/03635465020300061701

[16] DasMahapatra P Chiauzzi E Pujol LM Los C Trudeau KJ Mediators and moderators of chronic pain outcomes in an online self-management program. Clin J Pain 2015;31:404–13. 24918473 10.1097/AJP.0000000000000125 PMC4262714

[17] Devilly GJ Borkovec TD Psychometric properties of the credibility/expectancy questionnaire. J Behav Ther Exp Psychiatry 2000;31:73–86. 11132119 10.1016/s0005-7916(00)00012-4

[18] Dworkin RH Turk DC Farrar JT Haythornthwaite JA Jensen MP Katz NP Kerns RD Stucki G Allen RR Bellamy N Carr DB Chandler J Cowan P Dionne R Galer B Hertz S Jadad AR Kramer LD Manning DC Martin S McCormick CG McDermott MP McGrath P Quessy S Rappaport BA Robbins W Robinson JP Rothman M Royal MA Simon L Stauffer JW Stein W Tollett J Wernicke J Witter J; IMMPACT. Core outcome measures for chronic pain clinical trials: IMMPACT recommendations. PAIN 2005;113:9–19. 15621359 10.1016/j.pain.2004.09.012

[19] Fordyce WE Behavioral methods for chronic pain and illness. St. Louis: CV Mosby, 1976.

[20] Freedland KE Mohr DC Davidson KW Schwartz JE Usual and unusual care: existing practice control groups in randomized controlled trials of behavioral interventions. Psychosomatic Med 2011;73:323–35. 10.1097/PSY.0b013e318218e1fb PMC3091006 21536837

[21] Grine D Smith TB Culturally adapted mental health interventions: a meta-analytic review. Psychotheraphy (Chic) 2006;43:531–48. 10.1037/0033-3204.43.4.531 22122142

[22] Heapy AA Higgins DM Goulet JL LaChappelle KM Driscoll MA Czlapinski RA Buta E Piette JD Krein SL Kerns RD Interactive voice response-based self-management for chronic back pain: the COPES noninferiority randomized trial. JAMA Intern Med 2017;177:765–73. 28384682 10.1001/jamainternmed.2017.0223 PMC5818820

[23] Hilgard ER Huilgard JR Hypnosis in the relief of pain. Los Altos: Kaufman, 1975.

[24] Institute of Medicine. Relieving pain in America: a blueprint for transforming prevention, care, education, and research. Washington: National Academies Press, 2011. 22553896

[25] Juni P Altman DG Egger M Systematic reviews in health care: assessing the quality of controlled clinical trials. BMJ 2001;323:42–6. 11440947 10.1136/bmj.323.7303.42 PMC1120670

[26] Kapitza KP Passie T Bernateck M Karst M First non-contingent respiratory biofeedback placebo versus contingent biofeedback in patients with chronic low back pain: a randomized, controlled, double-blind trial. Appl Psychophysiol Biofeedback 2010;35:207–17. 20237953 10.1007/s10484-010-9130-1

[27] Kennedy-Martin T Curtis S Faries D Robinson S Johnston J A literature review on the representativeness of randomized controlled trial samples and implications for the external validity of trial results. Trials 2015;16:495. 26530985 10.1186/s13063-015-1023-4 PMC4632358

[28] Kuhajda MC Thorn BE Haskins SW Day MA Cabbil CM Literacy and cultural adaptations for cognitive behavioral therapy in a rural pain population. Transl Behav Med 2011;1:216–23. 24073046 10.1007/s13142-011-0026-2 PMC3717643

[29] Masheb RM Kerns RD Lozano C Minkin MJ Richman S A randomized clinical trial for women with vulvodynia: cognitive-behavioral therapy vs. supportive psychotherapy. PAIN 2009;141:31–40. 19022580 10.1016/j.pain.2008.09.031 PMC2728361

[30] Moher D Hopewell S Schulz KF Montori V Gøtzsche PC Devereaux PJ Elbourne D Egger M Altman DG CONSORT 2010 Explanation and Elaboration: updated guidelines for reporting parallel group randomised trials. J Clin Epidemiol 2010;63:e1–e37. 20346624 10.1016/j.jclinepi.2010.03.004

[31] Mohr DC Spring B Freedland K Beckner V Arean P Hollon SD Ockene J Kaplan R The selection and design of control conditions for randomized controlled trials of psychological interventions. Psychother Psychosom 2009;78:275–84. 19602916 10.1159/000228248

[32] Murphy JL Cognitive behavioral therapy for chronic pain among veterans: therapist manual. Washington: Department of Veterans Affairs, 2016.

[33] Okie S A flood of opioids, a rising tide of deaths. N Engl J Med 2010;363:1981–5. 21083382 10.1056/NEJMp1011512

[34] Onken LS Blaine JD Battjes R Behavioral therapy research: a conceptualization of a process. In: Henngler SW Amentos R, editors. Innovative approaches from difficult to treat populations. Washington: American Psychiatric Association Press, 1997 p. 477–85.

[35] Probst P Grummich K Heger P Zaschke S Knebel P Ulrich A Büchler MW Diener MK Blinding in randomized controlled trials in general and abdominal surgery: protocol for a systematic review and empirical study. Syst Res 2016;5:48. 10.1186/s13643-016-0226-4 PMC4806514 27012940

[36] Reid MC Otis J Barry LC Kerns RD Cognitive-behavioral therapy for chronic low back pain in older persons: a preliminary study. Pain Med 2003;4:223–30. 12974821 10.1046/j.1526-4637.2003.03030.x

[37] Resnick B Bellg AJ Borrelli B Defrancesco C Breger R Hecht J Sharp DL Levesque C Orwig D Ernst D Ogedegbe G Czajkowski S Examples of implementation and evaluation of treatment fidelity in the BCC studies: where we are and where we need to go. Ann Behav Med 2005;29(suppl):46–54. 15921489 10.1207/s15324796abm2902s_8

[38] Robb SL Burns DS Docherty SL Haase JE Ensuring treatment fidelity in a multi-site behavioral intervention study: implementing NIH Behavior Change Consortium recommendations in the SMART trial. Psychooncology 2011;20:1193–201. 22012943 10.1002/pon.1845 PMC3198011

[39] Rounsaville BJ Carroll KM Onken LS A stage model of behavioral therapies research: getting started and moving on from Stage I. Clin Psychol Sci Prac 2001;8:133–42.

[40] Sabate E Adherence to long-term therapies: evidence for action. Geneva: World Health Organization, 2003.

[41] Schulz KF Grimes DA Blinding in randomised trials: hiding who got what. Lancet 2002;359:696–700. 11879884 10.1016/S0140-6736(02)07816-9

[42] Schwartz CE Chesney MA Irvine MJ Keefe FJ The control group dilemma in clinical research: applications for psychosocial and behavioral medicine trials. Psychosom Med 1997;59:362–71. 9251155 10.1097/00006842-199707000-00005

[43] Taylor AM Phillips K Patel KV Turk DC Dworkin RH Beaton D Clauw DJ Gignac MA Markman JD Williams DA Bujanover S Burke LB Carr DB Choy EH Conaghan PG Cowan P Farrar JT Freeman R Gewandter J Gilron I Goli V Gover TD Haddox JD Kerns RD Kopecky EA Lee DA Malamut R Mease P Rappaport BA Simon LS Singh JA Smith SM Strand V Tugwell P Vanhove GF Veasley C Walco GA Wasan AD Witter J Assessment of physical function and participation in chronic pain clinical trials: IMMPACT/OMERACT recommendations. PAIN 2016;157:1836–50. 27058676 10.1097/j.pain.0000000000000577 PMC7453823

[44] Tharp RG Cultural diversity and treatment of children. J Cons Clin Psychol 1991;59:799–812. 10.1037//0022-006x.59.6.799 1774365

[45] The Office of the Assistant Secretary for Health at the U.S. Department of Health and Human Services. National pain strategy. Washington: Department of Health and Human Services, 2016.

[46] Thieme K Mathys M Turk DC Evidenced-based guidelines on the treatment of fibromyalgia patients: are they consistent and if not, why not? Have effective psychological treatments been overlooked? J Pain 2017;18:747–56. 28034828 10.1016/j.jpain.2016.12.006

[47] Thorn BE Day MA Burns J Kuhajda MC Gaskins SW Sweeney K McConley R Ward LC Cabbil C Randomized trial of group cognitive behavioral therapy compared with a pain education control for low-literacy rural people with chronic pain. PAIN 2011;152:2710–20. 21920668 10.1016/j.pain.2011.07.007 PMC3215913

[48] Turk DC Dworkin RH Allen RR Bellamy N Brandenburg N Carr DB Cleeland C Dionne R Farrar JT Galer BS Hewitt DJ Jadad AR Katz NP Kramer LD Manning DC McCormick CG McDermott MP McGrath P Quessy S Rappaport BA Robinson JP Royal MA Simon L Stauffer JW Stein W Tollett J Witter J Core outcome domains for chronic pain clinical trials: IMMPACT recommendations. PAIN 2003;106:337–45. 14659516 10.1016/j.pain.2003.08.001

[49] Turk DC Dworkin RH Revicki D Harding G Burke LB Cella D Cleeland CS Cowan P Farrar JT Hertz S Max MB Rappaport BA Identifying important outcome domains for chronic pain clinical trials: an IMMPACT survey of people with pain. PAIN 2008;137:276–85. 17937976 10.1016/j.pain.2007.09.002

[50] Turk DC Fillingim RB Ohrbach R Patel KV Assessment of psychosocial and functional impact of chronic pain. J Pain 2016;17(9 suppl):T21–T49. 27586830 10.1016/j.jpain.2016.02.006

[51] Turk DC Meichenbaum D Genest M Pain and behavioral medicine: a cognitive-behavioral perspective. New York: Guilford Press, 1983.

[52] Turk DC Wilson HD Cahana A Treatment of chronic non-cancer pain. Lancet 2011;377:2226–35. 21704872 10.1016/S0140-6736(11)60402-9

[53] Turner JA Holtzman S Mancl L Mediators, moderators, and predictors of therapeutic change in cognitive-behavioral therapy for chronic pain. PAIN 2007;127:276–86. 17071000 10.1016/j.pain.2006.09.005

[54] U.S. Department of Health and Human Services, Food and Drug Administration, Center for Drug Evaluation and Research (CDER). Draft guidance: guidance for industry analgesic indications: developing drug and biological products. 2014 Available at: https://www.fda.gov/downloads/drugs/guidancecomplianceregulatoryinformation/guidances/ucm384691.pdf. Accessed August 7, 2017.

[55] Vlaeyen JW Linton SJ Fear-avoidance model of chronic musculoskeletal pain: 12 years on. PAIN 2012;153:1144–7. 22321917 10.1016/j.pain.2011.12.009

[56] Wells PE Frampton V Bowsher D Pain management by physiotherapy. Oxford: Butterworth Heinemann, 1994.

[57] Wong EL Leung PC Zhang L Placebo acupuncture in an acupuncture clinical trial. How good is the blinding effect? J Acupunct Meridian Stud 2015;8:40–3. 25660443 10.1016/j.jams.2014.10.010

[58] Yates SL Morley S Eccleston C de C Williams AC A scale for rating the quality of psychological trials for pain. PAIN 2005;117:314–25. 16154704 10.1016/j.pain.2005.06.018

